# ﻿Temporal monitoring of genetic diversity in aquatic insects: a pilot study in the Bavarian Forest National Park

**DOI:** 10.3897/zookeys.1263.147797

**Published:** 2025-12-10

**Authors:** Jan Simon Stark, Oskar Schröder, Jörg Müller, Linda Seifert, Steffen U. Pauls

**Affiliations:** 1 Senckenberg Research Institute and Natural History Museum Frankfurt/M, Senckenberganlage 25, 60325 Frankfurt am Main, Germany Senckenberg Research Institute and Natural History Museum Frankfurt/M Frankfurt am Main Germany; 2 Institute for Insect Biotechnology, Justus-Liebig-University, Heinrich-Buff-Ring 26-32, 35392 Gießen, Germany Justus-Liebig-University Gießen Germany; 3 Department of River Ecology, Helmholtz Center for Environmental Research, Brückstrasse 3a, 39114 Magdeburg, Germany Department of River Ecology, Helmholtz Center for Environmental Research Magdeburg Germany; 4 Conservation and Research Department at the Bavarian Forest National Park, Freyunger Str. 2, 94481 Grafenau, Germany Conservation and Research Department at the Bavarian Forest National Park Grafenau Germany; 5 Field Station Fabrikschleichach, Chair of Conservation Biology and Forest Ecology, Biocenter, University of Würzburg, Glashüttenstraße 5, 96181 Rauhenebrach, Germany Biocenter, University of Würzburg Rauhenebrach Germany

**Keywords:** Ephemeroptera, global climate change, intraspecific genetic variation, microsatellites, Plecoptera, population structure, temporal genomics, Trichoptera

## Abstract

Temporal monitoring of genetic diversity within species is crucial for incorporating intraspecific genetic variation into global change monitoring. This pilot study aims to establish and validate a methodology for long-term genetic monitoring of aquatic insects in the Bavarian Forest National Park. Over seven years, the genetic diversity and spatiotemporal population structure of the mayfly *Baetis alpinus*, the stonefly *Brachyptera seticornis*, and the caddisfly *Drusus discolor* were investigated within three rivers in the national park. Utilising nuclear microsatellite analysis across nine loci for each species, 735 *B. alpinus*, 295 *B. seticornis*, and 193 *D. discolor* individuals were genotyped to assess local population structures in space and time. Our results revealed distinct spatial genetic patterns in *B. alpinus*, while *B. seticornis* and *D. discolor* lacked spatial genetic structure, indicating maintained gene flow throughout the populations within the national park. *B. alpinus* exhibited site-specific temporal changes, while *B. seticornis* and *D. discolor* showed stable genetic structures over the study period. These findings demonstrate the ability of this system to detect subtle genetic shifts over time. Furthermore, the results emphasise that environmental changes act species-specifically, highlighting the importance of species-level approaches in ecological studies. Continued monitoring and expanded analyses will further our understanding of ecological and population dynamics in changing environments. In light of our preliminary data, it is posited that neutral intraspecific genetic variation is a key indicator of the impacts of global change on freshwater species as it is often among the first aspects of biodiversity to reflect the consequences of changing environmental conditions in a species.

## ﻿Introduction

Genetic variation within a species serves as the foundational component of biodiversity (May 1994). Intraspecific genetic diversity is not only the basis of all evolutionary processes, it also underpins population fitness and is essential for maintaining species adaptability (Reed and Frankham 2003; Des Roches et al. 2018). A decline in genetic variation may pose a threat to a species’ fitness and adaptation to environmental disturbances (Gienapp et al. 2008). This is particularly critical in the context of global climate change (GCC), where genetic variation is essential for species to adapt to rapidly changing environmental conditions (Leigh et al. 2019) and shifts might indicate adaption of species to altered environmental conditions. Comprehensive studies on the overarching impacts of GCC have predominantly focused on ecosystem and species-level responses, often overlooking the consequences on intraspecific genetic variation (Bálint et al. 2011; Pauls et al. 2013; Des Roches et al. 2021; Lyam et al. 2022). This oversight underscores a critical research gap in the understanding of the multifaceted repercussions of global environmental change (including GCC) on the genetic diversity of populations.

Temporal variations in genetic structure offer a dynamic perspective on the consequences of environmental change on populations. However, temporal genomic studies remain underrepresented in global change monitoring (Jensen and Leigh 2022). Similarly, while spatial population genetic structure has been documented in several stream insects (e.g., Monaghan et al. 2002; Finn et al. 2006; Taubmann et al. 2011; Alp et al. 2012; Schröder et al. 2022), studies on temporal dynamics are lacking.

Montane ecosystems, which are known for their high levels of biodiversity, are especially vulnerable to the impacts of GCC, as species migrating upslope in response to warming face shrinking habitats (La Sorte and Jetz 2010; McCain and Grytnes 2010). In the Bavarian Forest National Park, a climate-sensitive zone has been identified between 1100 and 1200 m a.s.l., where temperature-driven shifts in species distributions might occur (Bässler et al. 2010). Cold-adapted species, in particular, are at risk of significant range contractions, which may lead to a substantial loss of genetic diversity (Moussalli et al. 2009; Bálint et al. 2011; Taubmann et al. 2011). This, in turn, may alter population connectivity, subsequently influencing the genetic structure (Chen et al. 2011).

This pilot study aims to establish a methodology for long-term genetic monitoring of freshwater invertebrates within the Bavarian Forest National Park (BFNP). By examining neutral genetic diversity and population structure of three aquatic insects, *Baetis alpinus* (Ephemeroptera), *Brachyptera seticornis* (Plecoptera), and *Drusus discolor* (Trichoptera), across a temporal scale, we seek to provide insights into species dispersal and short-term population dynamics. *D. discolor* is cold stenotherm, highly specialised for cold-water habitats, making it particularly vulnerable to warming. *B. alpinus* is primarily cold-adapted but exhibits a broader ecological range compared to *D. discolor*. In contrast, *B. seticornis* is eurythermic, thriving across a wider range of thermal conditions. This enables the comparison of population responses among species with differing thermal sensitivities. Specifically, this study (i) investigates the presence of genetic differentiation or gene flow among regional populations of these species across and within the three distinct rivers of the BFNP. Our study also (ii) examines the potential differences in population genetic structure across the three regionally co-distributed species. Finally, the research (iii) probes into the temporal shifts in their population genetic structures, focusing on interannual variations and examining how the genetic diversity of individual populations within each species has evolved over the study period.

To achieve these objectives, we employed nuclear microsatellite analysis, which is particularly valuable for detecting neutral genetic variations within and between populations due to their high mutation rates, co-dominant inheritance, and polymorphic nature (Abdul-Muneer 2014). Non-neutral evolution of microsatellites has been documented, but the markers used here have not shown any evidence for this in a previous spatial, non-temporal analysis (Schröder et al. 2022). Despite advances in genome-wide approaches, microsatellites remain a practical and cost-effective tool for population genetic studies, offering high resolution for detecting fine-scale genetic structure over space and time while allowing consistency in long-term monitoring efforts. While this method does not allow identifying adaptive genetic responses to environmental changes, it provides essential insights into population-level dynamics by focusing on neutral genetic changes. This approach enables a comprehensive assessment of the spatial and temporal genetic structure of freshwater invertebrates within the BFNP, thereby establishing a robust framework for long-term genetic monitoring that will improve our understanding of populations’ responses to environmental changes.

## ﻿Materials and methods

### ﻿Study sites and sampling

**Figure 1.**
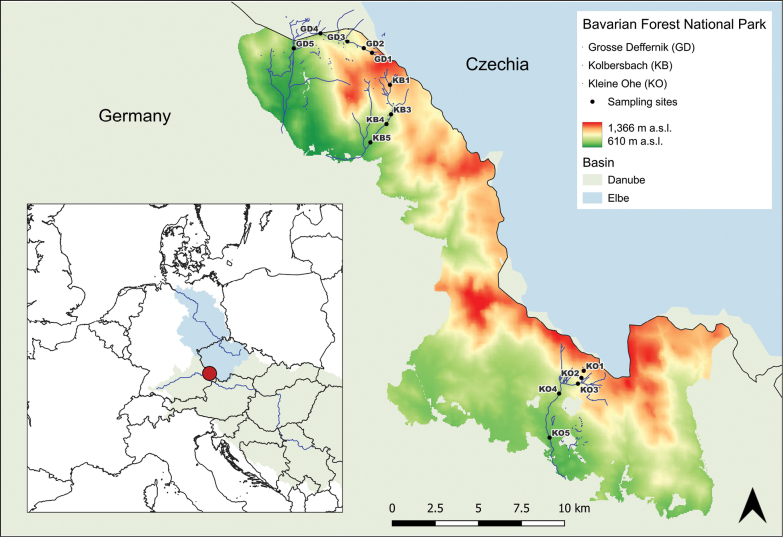
Map of sampling sites within the Bavarian Forest National Park in eastern Bavaria, Germany. The red dot on the inset map indicates the location of the national park. Sampling sites are located at the Kolbersbach (KB), Kleine Ohe (KO), and Grosse Deffernik (GD) at different altitudes within the national park.

This study was conducted in the Bavarian Forest National Park in southeastern Germany, along the Czech border (Fig. 1). The BFNP was established in 1970 as Germany’s first national park (Bibelriether 2004). It spans 249.45 km^2^ in the Bavarian Forest (NPBW 2023). Together with the neighbouring Šumava National Park in the Czech Republic, it forms the largest contiguous forest reserve in Central Europe (NPBW 2023). The Bavarian Forest is predominantly situated within the catchment area of the Danube River systems and partially within the Elbe River systems in the northeast (Bierschenk et al. 2018).

For this study, we collected larvae from three streams between 2016 and 2023 along elevation gradients, ranging from 700 m to 1100 m above sea level (a.s.l.). The Große Deffernik (GD) and the Kolbersbach (KB) in the north and the Kleine Ohe (KO) in the south of the BFNP (Fig. 1). A total of seven sampling events were conducted. Geographical coordinates of the sampling sites are provided in Suppl. material 1: table S1. All streams are tributaries of the Danube River system. Sampling was conducted annually, from late April to early May. Larvae were collected from 50 m long sections upstream of each sampling site using kick nets and by hand collection. Up to 25 specimens per target species and site were obtained and promptly stored in 96% ethanol at < 4 °C. Sites within each stream were spaced at 200 m intervals in elevation, starting from 700 m a.s.l. For 2016 and 2018, additional sites at 800 m and 1100 m a.s.l. were sampled. Specimens were classified morphologically under a stereoscope using the keys of Eiseler (2005) for *B. alpinus*, Zwick (2004) for *B. seticornis*, and Waringer and Graf (2011) for *D. discolor*. In some cases, low numbers of individuals were supplemented from a general benthic invertebrate monitoring scheme that encompasses some of the same sites.

### ﻿Study species

*Baetis alpinus* Pictet, 1843 (Ephemeroptera: Baetidae) is a common mayfly that is widely distributed in alpine mountain streams (Humpesch 1979; Breitenmoser-Würsten and Sartori 1995). It is characterised by a complex of reproductively isolated cryptic lineages, two of which have been documented in the Swiss Alps (Leys et al. 2016). Only one cryptic lineage (Lineage A) is found in the Bavarian Forest (Fig. 4; Schröder et al. 2022). This species occurs in stony, fast-flowing streams located between 200 m and at least 2600 m a.s.l. (Leys et al. 2017). Larvae are scrapers that feed on diatoms and detritus adhering to substrate surfaces (López-Rodríguez et al. 2009). This species displays notable life cycle plasticity, characterised by varying voltinism (semi-, uni-, or multivoltine) and emergence timing influenced by environmental drivers, primarily temperature (Lavandier 1988; Breitenmoser-Würsten and Sartori 1995; Kukula 1997; Knispel et al. 2006; López-Rodríguez et al. 2009).

*B. alpinus* is primarily adapted to cold water conditions, with an optimal thermal range of 4–8 °C for its development, although it demonstrates a degree of thermal plasticity within this range (Humpesch 1979; Ward and Stanford 1982; Knispel et al. 2006). Temperature dependence affects the developmental rates of both larval and egg stages (Humpesch 1979; Knispel et al. 2006). The adults are short-lived, but emergence can extend over several months and is often asynchronous (Humpesch 1979; Kukula 1997). Mating occurs in swarms formed by males, into which females fly to mate (Brittain 1982). Adult *B. alpinus*, particularly females, exhibit upstream flight behaviour that helps counteract the downstream drift of larvae (Thomas 1975; Lavandier 1982; Schmidt et al. 1995). This behaviour can enhance connectivity among populations, particularly in fragmented habitats, where geographical barriers might disrupt gene flow (Monaghan et al. 2001).

The stonefly *Brachyptera seticornis* Klapálek, 1902 (Plecoptera: Taeniopterygidae) is widespread in montane and submontane streams in central, eastern, and southern Europe (Küttner et al. 2008; Bojková et al. 2011; Tyufekchieva et al. 2012). Following Kermek et al. (2024), it appears to represent a well-defined lineage with mitochondrial substructure in south-eastern European mountains. It occurs in fast-flowing, stony, and crystalline streams (Küttner et al. 2008; Krno et al. 2021) and is adapted to cooler water temperatures (Haidekker and Hering 2009). Larvae are specialised scrapers that feed on benthic algae and stone biofilms (Dangles 2002; Krno et al. 2013). The adult lifespan of *Brachyptera* ranges from three to four weeks (Küttner et al. 2008). Upstream migration to compensate for downstream larval drift was observed in adults of the closely related species, *Brachyptera risi* (Morton 1896) (Plecoptera) (Madsen et al. 1973). Generally, the movement pattern of *B. risi* can be highly variable, with adults travelling considerable distances from their natal watercourse despite its limited flying capabilities (Madsen et al. 1973). Based on the related trait knowledge, it is very plausible that *B. seticornis* behaves similarly.

*Drusus discolor* Rambur, 1842 (Trichoptera: Limnephilidae) is a caddisfly that is predominantly distributed across European mountain streams. Multiple locally restricted evolutionary lineages have been identified for this species across Europe (Pauls et al. 2006, 2009; Geismar et al. 2015). It occurs in cold headwater streams in central European mountains above 600 m a.s.l., in high alpine streams exceeding 2000 m in the Alps, and in southern European mountain ranges (Pauls et al. 2006). The larvae inhabit fast-flowing streams. Early instar larvae are shredders feeding on periphyton, whereas the last instars are filter-feeding carnivores that eat periphyton and invertebrates (Lavandier 1992). The life cycle is uni- to semi-voltine with an emergence period lasting several months in the summer (Lavandier 1992). *D. discolor* is adapted to cold waters, with mean annual temperatures slightly above 0 °C and summer maxima not exceeding 10 °C (Lods-Crozet et al. 2001). The movement of larvae in streams is limited to either upstream or downstream directions, whereas adult dispersal is restricted to a few kilometres (e.g., Geismar et al. 2015), as observed in other caddisfly species (Sode and Wiberg-Larsen 1993; Kovats et al. 1996; Collier and Smith 1998).

### ﻿Laboratory methods and genotyping

Genomic DNA was extracted from the thorax of all specimens using the HotSHOT method (Truett et al. 2000). The remaining parts were deposited as vouchers in the collections of the Senckenberg Natural History Museum, Frankfurt/M, Germany. Specifically, 50 µL of Alkaline Lysis Reagent (25 mM NaOH, 0.2 mM EDTA) was added to the tissue and incubated at 95 °C for 30 min. After incubation, 50 µL of Neutralisation Reagent (40 mM Tris-HCl) was added.

The microsatellite loci targeted for *B. alpinus* in this study were Ba_c1, Ba_c2, Ba_c3, Ba_c4, Ba_g1, Ba_g2, Ba_g4, Ba_g5, and Ba_g6 (Leys et al. 2017; Suppl. material 1: table S2). For *B. seticornis*, the loci BS_11, BS_16, BS_18, BS_34, BS_35, BS_36, BS_43, BS_52, and BS_53 were used (Schröder et al. 2022; Suppl. material 1: table S3). *D. discolor* specimens were genotyped at loci DD_03, DD_05, DD_12, DD_17, DD_31, DD_52, DD_59, DD_61, and DD_66 (Pauls et al. 2007; Geismar et al. 2011; Suppl. material 1: table S4). Loci selection for each species for genotyping was based on the amplification performance and signal quality of the raw data.

Microsatellite loci were amplified using the Qiagen Type-it Microsatellite PCR Kit, according to the manufacturer’s protocol. For the multiplex polymerase chain reactions (PCRs), 10 μl reactions were prepared with 1 μl template DNA containing 10 ng genomic DNA. In instances of low DNA yield, particularly in some years for *D. discolor*, 5 ng genomic DNA was used. DNA concentration was measured using the Qubit 1x dsDNA-HS Assay-Kit (Thermo Fisher Scientific). Following the approach of Blacket et al. (2012), a three-primer PCR method was adopted that uses a fluorescently labelled universal primer paired with modified locus-specific primers having 5′ universal primer sequence tails. The number of cycles varied between 28 and 30, depending on the DNA content and sequencing performance. After amplification, the reactions were diluted and analysed using a 3730XL DNA Analyser (Applied Biosystems) located at the Senckenberg Biodiversity and Climate Research Centre Laboratory Centre, Frankfurt/M, Germany. Subsequently, fragment analysis and allele calling were performed using GeneMarker v. 3.0.0 (Softgenetics).

### ﻿Population genetic structure and genetic diversity

Population genetic structure and genetic diversity metrics were initially calculated using ARLEQUIN v. 3.5.2.2 (Excoffier and Lischer 2010). This included locus-by-locus analysis of molecular variance (AMOVA) to assess for differences in genetic structure and diversity among years, streams, and sites within streams (α = 0.05, 1000 permutations). The fixation index (F_ST_), which quantifies the effect of subpopulations compared to the total population, was used in pairwise comparisons to further assess these differences (α = 0.05, 1000 permutations). To adjust significance levels for multiple comparisons, false discovery rate (FDR) corrections (Benjamini and Hochberg 1995) were applied to the F_ST_ p-values. Additional summary statistics for genetic diversity, such as observed heterozygosity (H_o_), expected heterozygosity (H_e_), F_ST_, the inbreeding coefficient relative to the subpopulation (F_IS_), allelic richness (A_R_), and total allelic richness (A_T_) were calculated per locus using FSTAT v. 2.9.4 (Goudet 2003). Null allele frequencies were estimated using FreeNA (Chapuis and Estoup 2007). Only specimens in which more than half of the loci were successfully amplified were included in the analyses.

The Bayesian clustering algorithm in STRUCTURE v. 2.3.4 (Pritchard et al. 2000) was used to infer the number of genetic clusters (K) present in the dataset to display population structure over the observation period, among streams, and among sites within streams. Ten independent runs for each value of K (ranging from 1–10) were performed, with a burn-in period of 10,000 steps, followed by 100,000 Markov Chain Monte Carlo (MCMC) iterations. The optimal number of clusters was determined using Structure Harvester Web v. 0.6.94 (Earl and vonHoldt 2012), which employs the Evanno method (Evanno et al. 2005). The Evanno method has been reported to favour lower K values, especially in hierarchical or subtle population structures (Puechmaille 2016). Thus, to refine the results and ascertain a more accurate estimation of ΔK, specimens were randomly removed from the dataset to equalise the sizes of the populations for analysis. This random removal was repeated ten times, and STRUCTURE analyses were conducted separately for each replicate. The subsampling procedure was applied only for ΔK estimation and not for other analyses. Data visualisation of the STRUCTURE outputs was performed using DISTRUCT v. 1.1 (Rosenberg 2004).

## ﻿Results

### ﻿Spatial and temporal genetic structure of *Baetis alpinus*

Of the *B. alpinus* specimens collected from eleven sites between 2016 and 2023, 735 were successfully genotyped. All microsatellite loci were polymorphic (Suppl. material 1: table S5). Estimated null allele frequency among all loci was 7.47%, varying between 1.09% (Ba_c1) and 21.32% (Ba_c3). For the full dataset, the best fitting number of clusters was estimated to be K = 2. STRUCTURE runs based on randomly subsampled datasets supported K = 3 in eight out of ten replicates and K = 2 in two, with K = 3 representing the most stable clustering result when accounting for unequal sample sizes.

**Figure 2.**
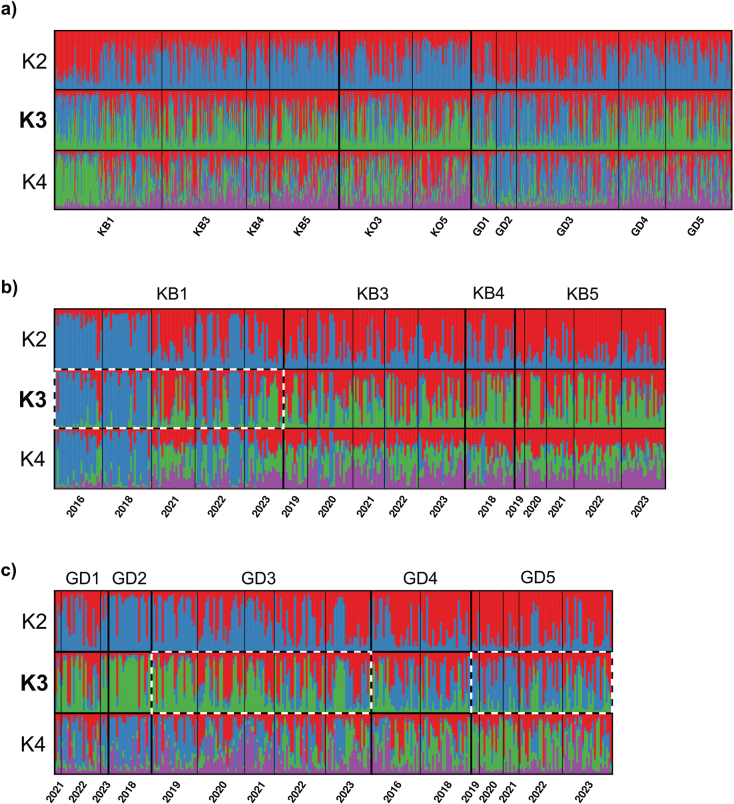
Population genetic structure of *B. alpinus* across sampling sites and years. **a.** Genetic structure for all sampling sites within the Kolbersbach (KB), the Kleine Ohe (KO), and the Grosse Deffernik (GD) across all years, *n* = 735; **b.** Genetic structure for all sampling sites in Kolbersbach (KB) separated by years from 2016-2023, *n* = 309; **c.** Genetic structure for all sampling sites in the in the Grosse Deffernik (GD) separated by years from 2016-2023, *n* = 282. Results were revealed by STRUCTURE. K = 3 represents the best fit of genetic clusters. Dashed rectangles indicate sites with distinct temporal shifts.

A spatial pattern was observed among the sites within streams, where the elevation gradient exhibited more genetic variation within streams than between streams (Fig. 2a). The AMOVA yielded similar results, revealing a higher percentage of variation among sites within streams than among streams (Suppl. material 1: table S6). Among all sampling sites, 41 out of 55 pairwise F_ST_ comparisons were significant (Suppl. material 1: table S7). Separate STRUCTURE analyses for each stream, encompassing sampling sites within streams and across years, highlighted both spatial and temporal genetic variations (Fig. 2b, c). The AMOVA results indicated limited differentiation among years across all streams (Suppl. material 1: table S8). No temporal genetic structure was evident in the Kleine Ohe. However, temporal changes were observed in the Kolbersbach, with particularly significant shifts at site KB1 (Fig. 2b). Additionally, distinct temporal shifts were evident at GD3 and GD5 in the Grosse Deffernik (Fig. 2c). In 2021, at sampling site KB1 in the Kolbersbach, the relative genetic cluster contributions showed greater divergence from all other years at KB1 (Fig. 2b). Pairwise F_ST_ comparisons revealed that for all three streams, both 2016 and 2018 exhibited slight yet significant differences from more recent years (Suppl. material 1: table S9). The most pronounced difference (F_ST_ = 0.016) was observed between 2016 and 2023. Overall, nine out of 21 comparisons were statistically significant (p < 0.05), which was further emphasised by the STRUCTURE analysis.

### ﻿Spatial and temporal genetic structure of *Brachyptera seticornis*

Of the *B. seticornis* specimens collected from eleven sites between 2018 and 2023, 295 were successfully genotyped. For 2021, due to the low number of sampled specimens (*n* = 11), limited data were available. All microsatellite loci were polymorphic (Suppl. material 1: table S10). Null allele frequency varied between 0.51% (BS_18) and 19.11% (BS_16), with 4.51% among all loci. For *B. seticornis*, the population structure was best described by a K value of two, applicable to both the full and the simulated dataset. All STRUCTURE runs with equalised sample sizes consistently supported K = 2 as the best fitting number of clusters.

**Figure 3.**
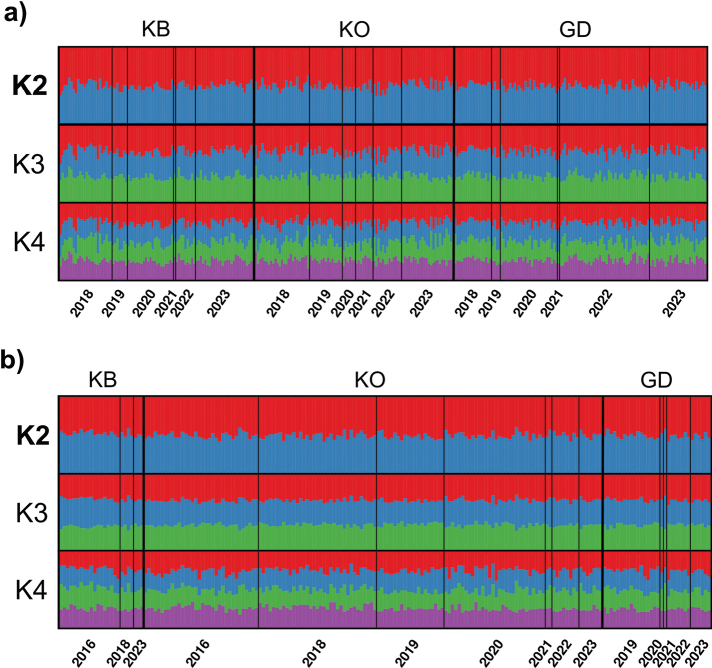
Population genetic structure of *B. seticornis* and *D. discolor* across sampling sites from 2016-2023. **a.** Genetic structure of *B. seticornis* (*n* = 295) for the Kolbersbach (KB), the Kleine Ohe (KO), and the Grosse Deffernik (GD); **b.** Genetic structure of *D. discolor* (*n* = 193) for the Kolbersbach (KB), the Kleine Ohe (KO), and the Grosse Deffernik (GD). Results were revealed by STRUCTURE. K = 2 represents the best fit of genetic clusters for both species.

Bayesian cluster analysis did not reveal any genetic differences among years, streams, or sites within streams (Fig. 3a), which is further corroborated by only two of 15 significant F_ST_ comparisons for all streams from 2018 to 2023 and no significant pairwise F_ST_ value for any of the 55 compared sampling sites (Suppl. material 1: tables S11, S12). The subsequent AMOVA also revealed no temporal or spatial patterns in this species (Suppl. material 1: tables S13, S14).

### ﻿Spatial and temporal genetic structure of *Drusus discolor*

Of the *D. discolor* specimens collected from six sampling sites between 2016 and 2023, 193 were successfully genotyped. *D. discolor* was found nearly exclusively from 900 m a.s.l. upwards, with one exception at sampling site GD5. Furthermore, the number of specimens collected declined during the observation period (cf. Fig. 5). Most microsatellite loci were polymorphic, except for locus DD_05, which was monomorphic across all sampled individuals (Suppl. material 1: table S15). The estimated null allele frequency among all loci was 5.53%, ranging from 0.09% (DD_05) to 19.30% (DD_61). For both the full and the simulated dataset, two genetic clusters were determined for *D. discolor*. All STRUCTURE replicates based on subsampled datasets supported K = 2.

Similar to *B. seticornis*, STRUCTURE analysis did not reveal any genetic differences among years, streams, or sites within streams (Fig. 3b). Subsequently, significant differentiation was detected in only 5 out of 21 pairwise F_ST_ comparisons for all streams between 2016 and 2023 (Suppl. material 1: table S16). In addition, two significant F_ST_ values were identified out of 15 comparisons among all sampling sites (Suppl. material 1: table S17). The AMOVA could not discern any temporal or spatial patterns within this species (Suppl. material 1: tables S18, S19).

## ﻿Discussion

### ﻿Diverse patterns of spatial genetic structure

Genetic diversity and population structure of three aquatic insect species, *Baetis alpinus*, *Brachyptera seticornis*, and *Drusus discolor*, were analysed in three streams within the Bavarian Forest National Park over a period of seven years between 2016 and 2023. For *B. alpinus*, a significant population structure was detected, characterised by spatial genetic variation across different elevations within individual streams. However, no variation was detected among the streams. On the other hand, no significant spatial population structure was observed for *B. seticornis* and *D. discolor* within the BFNP.

The population genetic structure observed in *B. alpinus* aligns with previously described spatially distributed patterns for this species in the Swiss alps (Monaghan et al. 2002; Leys et al. 2017) and within the BFNP (Schröder et al. 2022). The observed spatial variation in genetic structure across different elevations indicates restricted gene flow among sites within streams. This restriction may be attributed to the short lifespan of *B. alpinus* imagines, which limits their ability to disperse, migrate, and mix with populations in other streams or at different elevations within the BFNP. Leys et al. (2017) similarly reported that life-history traits, such as short lifespans and limited dispersal, contribute to genetic differentiation in this species. This pattern was also observed in other aquatic insect species, particularly in isolated populations (Rutschmann et al. 2017). Consequently, this may result in lower migration rates between sites, as reflected in the distinct genetic structures at different spatial sites. Thus, the observed differences in genetic structure, even within spatially proximate areas, could potentially be explained by the patchy recruitment hypothesis (Bunn and Hughes 1997). This hypothesis postulates that sporadic successful recruitment events, driven by a limited number of successfully ovipositing females per site, lead to transient and dynamic population structures, a pattern observed in Australian baetid species (Schmidt et al. 1995; Hughes et al. 2003). However, the less fluctuating population structure of *B. alpinus* within the BFNP over the seven-year study period contrasts with the transient, dynamic population structures proposed by patchy recruitment. The observed genetic divergence at sampling site KB1 in 2021 could be indicative of colonisation effects, introducing distinct genetic material and contributing to genetic differentiation from other years at this site.

**Figure 4.**
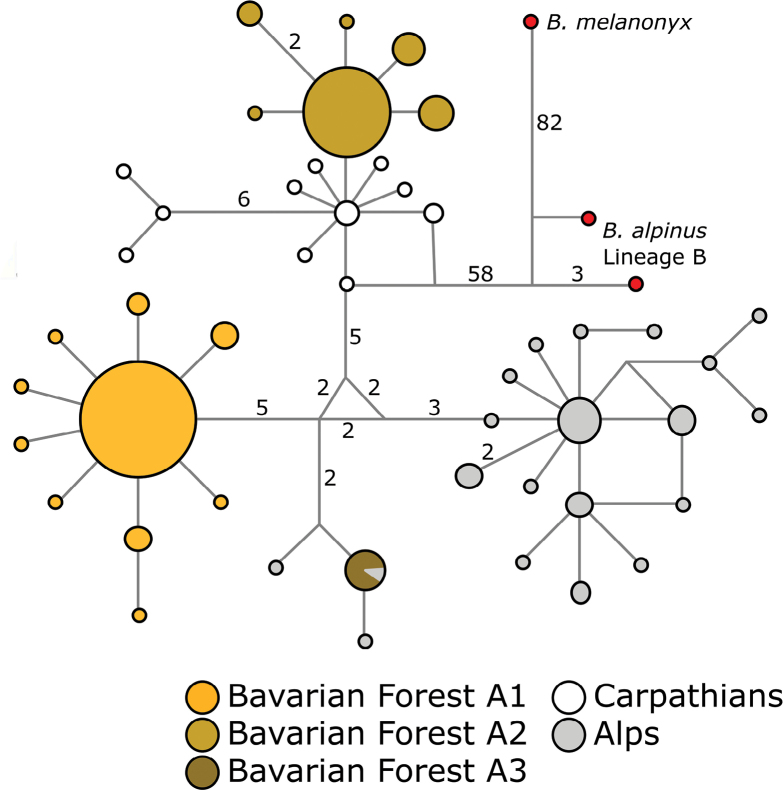
Median-joining haplotype network for *B. alpinus* (lineage A). The network is based on a 367-bp cytochrome-c-oxidase subunit I sequence from 248 individuals, including specimens sampled in the Bavarian Forest National Park in 2016, 2018, and 2019, and from two other European mountain ranges. Numbers on branches denote base pair changes (default = 1), and circle size represents the number of individuals with that haplotype. Adapted from Schröder et al. (2022).

Within the BFNP, the *B. alpinus* species complex is represented by a single lineage (Fig. 4, lineage A), which encompasses different sublineages, as identified by Schröder et al. (2022). In their study, haplotype networks were constructed using NETWORK v. 10 based on a 367-bp alignment of mitochondrial COI sequences. The haplotypes were derived from specimens collected over three years (2016, 2018, and 2019) across seven streams within the BFNP, including the three streams featured in this study, as well as from two other mountain ranges. The dominant haplogroup A1 is autochthonous, whereas haplogroup A2 suggests an eastern lineage invasion from the Carpathians, and haplogroup A3 may hint at an introgression from the Alps. Each haplogroup, with its distinct geographical and possibly ecological affinities, could contribute to the spatial genetic heterogeneity observed within the BFNP. Moreover, the spatial distribution of genetic variation may be influenced by interactions among these lineages, which reflect both historical invasions and potential contemporary introgressions.

In contrast to *B. alpinus*, *B. seticornis* exhibited no significant spatial population structure within the BFNP, suggesting a panmictic population where individuals are likely to mate randomly across spatial scales within this region. This lack of a discernible population structure might reflect greater connectivity between populations of *B. seticornis*, thus suggesting substantial gene flow among populations within the BFNP. Similar observations have been reported in a previous study conducted on this species within the BFNP (Schröder et al. 2022), as well as for other stonefly species, where a lack of regional population structure was observed (Elbrecht et al. 2014; Malison et al. 2022). Compared to *B. alpinus*, *B. seticornis* has a longer adult lifespan which may allow for extended flight periods during the winged adult stage, facilitating enhanced connectivity both within and across streams, hence leading to genetic homogenisation.

Individuals of *D. discolor* were predominantly found within headwaters. Adaptation to headwater habitats may be associated with cooler temperature regimes and microhabitat characteristics of these areas, indicating specialisation for headwaters. Similar to *B. seticornis*, *D. discolor* showed no significant spatial population structure within the BFNP. Such consistency in population structure implies a high degree of gene flow among *D. discolor* populations within the BFNP, demonstrating the species’ capability for adult dispersal across headwaters within this region. This observation agrees with the Headwater Model (Finn et al. 2007), which suggests genetic connectivity for headwater specialists among adjacent headwaters and even between catchments through overland dispersal. In the same vein, successful dispersal was described for this species even across multiple catchments within German mountain ranges (Geismar et al. 2015), which further supports the hypothesis of effective overland dispersal across streams within the BFNP. In addition, extended adult flight periods could further maintain gene flow across populations within the BFNP (Kelly et al. 2001). At larger spatial scales, *D. discolor* populations have been shown to exhibit no significant genetic structure within mountain ranges (Pauls et al. 2006; Lehrian et al. 2009), a pattern that is also observed within the BFNP.

### ﻿Temporal genetic structure dynamics

Over the seven-year study period, the population genetic structure of *B. alpinus* exhibited notable site-specific temporal changes, particularly at sites KB1 in the Kolbersbach and GD3 and GD5 in the Grosse Deffernik. In contrast, the stable genetic structures of *B. seticornis* and *D. discolor* across all sites and years indicate that there were no significant temporal shifts or interannual variations. These observations indicate that the monitoring system is sufficiently variable to detect genetic shifts when they occur, e.g., in *B. alpinus*. By design, observable shifts reflect changes in neutral genetic diversity and are not directly attributable to a specific environmental driver. But with continued monitoring our approach will reveal putative consequences of environmental change on the long-term dynamics of genetic diversity and population structure intraspecific diversity in the target species.

The hitherto seven-year timescale is admittedly limited and insufficient to observe significant changes in genetic structure. This makes it particularly difficult to predict future trends. Nevertheless, several factors could explain the observed patterns. For *B. seticornis* and *D. discolor*, the genetic structure showed stability across all sites and years. This stability could be due to consistent gene flow among populations, leading to short-term temporal stability.

In contrast, *B. alpinus* exhibited site-specific temporal changes. These changes could be influenced by local population dynamics. Genetic drift in smaller subpopulations can lead to detectable shifts in genetic diversity over time (Demandt 2010). The lower migration rates observed in *B. alpinus* might contribute to these site-specific changes, as reduced gene flow can cause genetic differences to become more pronounced at specific sites. No bottleneck or founder events occurred that could have significantly impacted the genetic structure of populations.

**Figure 5.**
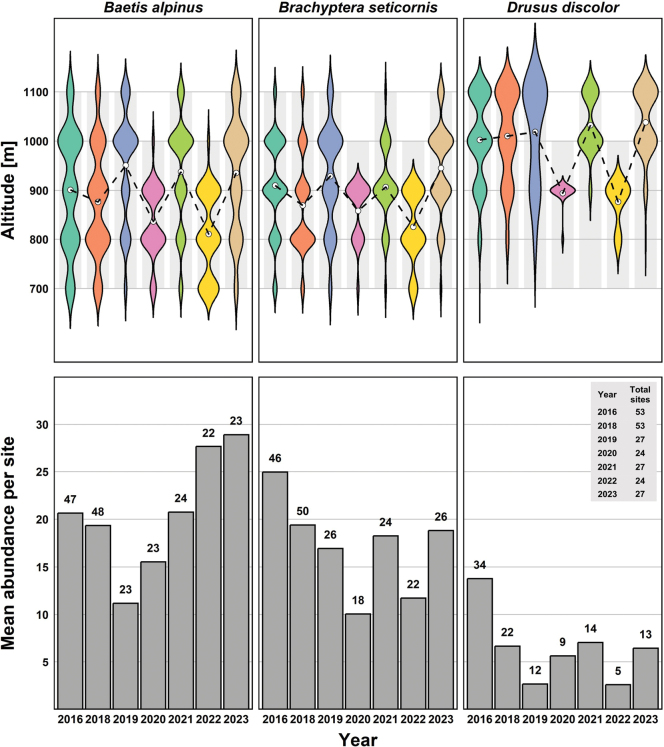
Annual distributions and abundances of *Baetis alpinus*, *Brachyptera seticornis*, and *Drusus discolor*. The upper panels display the altitude distributions of each species, with violin plots showing the density of individuals across altitudes (700 m, 800 m, 900 m, 1000 m, 1100 m) from 2016 to 2023. Dashed lines indicate mean altitudes, and shaded areas represent the altitudinal ranges sampled in each year. The lower panels represent the mean annual abundances of individuals per sampling site, collected through Multi-Habitat-Sampling in the BFNP. The numbers above each bar represent the number of sampling sites where each species was found in that year. The total number of sampling sites for each year is provided in a separate legend.

Interestingly, over the course of the study period, there was a notable decline in the number of *D. discolor* individuals collected across all sites, indicating a potential decrease in the population of this species within the studied streams. To further explore this observation, we examined annual Multi-Habitat-Sampling data from eight streams within the BFNP, including the three streams that are the focus in this study (Fig. 5). The results reveal varying patterns. While the abundance of *B. alpinus* seems to be increasing in recent years following a dip in 2019, the other two species rather show signs of decline since the initial sampling in 2016. This seems particularly evident in *D. discolor*, where the number of specimens per site has been below 20 specimens per site since 2019. Especially the low elevation sites seem to produce very few specimens of *D. discolor*. While this pattern is definitely not conclusive, this may be resulting from the species migrating uphill and upstream under climate warming as predicted in previous studies (e.g., Bálint et al. 2011; Domisch et al. 2011; Sauer et al. 2011). However, the current data are insufficient to confirm this trend.

The exploration of temporal population genetic structure, especially in aquatic insects and other stream invertebrates, provides vital insights into population dynamism, thus enhancing the understanding of the effects of environmental change on biodiversity. For example, intraspecific genetic diversity provides a richer lens for studying GCC impacts, as populations within species exhibit varied responses to changing conditions (Pauls et al. 2013). This study underscores the importance of including temporal genetic monitoring in conserving genetic variation. Global change monitoring often prioritises species biodiversity and abundance, while intraspecific genetic diversity, which is crucial for species adaptation and evolution, is commonly overlooked (Bálint et al. 2011; Pauls et al. 2013; Leigh et al. 2019; Laikre et al. 2020). Cold-water adapted aquatic invertebrates, such as those examined in this study, constitute paradigmatic examples of species that are exceptionally sensitive to global warming and may be among the most vulnerable to the impacts of a warming climate (Hering et al. 2009; Sauer et al. 2011).

## ﻿Conclusions

The established monitoring system enabled effective and straightforward long-term genetic monitoring in the BFNP, with the capacity to detect subtle shifts in intraspecific genetic diversity that may reflect the impacts of GCC on aquatic insect populations. By focusing on neutral genetic markers, it is possible to detect broader consequences of environmental changes on populations. Although neutral markers do not directly reveal adaptive genetic changes, they remain invaluable for monitoring real-world biodiversity trends, where complex interactions often hinder the identification of specific causal links. While the present dataset offers an initial perspective, extensive long-term genetic monitoring is crucial to assess species-to-species responses to environmental shifts, particularly for threatened montane aquatic invertebrates facing the challenges of global environmental change. This is vital since appropriate conservation actions rely on continuous responses to environmental change (Jensen and Leigh 2022). Establishing a foundational genetic baseline, could pave the way for subsequent in-depth temporal genomic analyses to decipher broad patterns and determinants of intraspecific genetic variation. And broadly incorporating genetic diversity into biomonitoring for environmental change is essential for detecting changes in populations before species are locally extirpated.

## ﻿Additional information

### Author ORCIDs

Jan Simon Stark https://orcid.org/0009-0006-3863-3841

Oskar Schröder https://orcid.org/0000-0001-8200-0669

Jörg Müller https://orcid.org/0000-0002-1409-1586

Steffen U. Pauls https://orcid.org/0000-0002-6451-3425

### Data availability

All of the data that support the findings of this study are available in the main text or Supplementary Information.

## Supplementary materials

Supplementary material 1 Supplementary information Data type docx https://binary.pensoft.net/file/1488928This dataset is made available under the Open Database License (http://opendatacommons.org/licenses/odbl/1.0/). The Open Database License (ODbL) is a license agreement intended to allow users to freely share, modify, and use this Dataset while maintaining this same freedom for others, provided that the original source and author(s) are credited.Jan Simon Stark, Oskar Schröder, Jörg Müller, Linda Seifert, Steffen U. Pauls

## References

[B1] Abdul-Muneer PM (2014) Application of microsatellite markers in conservation genetics and fisheries management: Recent advances in population structure analysis and conservation strategies. Genetics Research International 691759: 1–11. 10.1155/2014/691759 PMC3997932 24808959

[B2] Alp M Keller I Westram AM Robinson CT (2012) How river structure and biological traits influence gene flow: A population genetic study of two stream invertebrates with differing dispersal abilities. Freshwater Biology 57(5): 969–981. 10.1111/j.1365-2427.2012.02758.x

[B3] Bálint M Domisch S Engelhardt CHM Haase P Lehrian S Sauer J Theissinger K Pauls SU Nowak C (2011) Cryptic biodiversity loss linked to global climate change. Nature Climate Change 1(6): 313–318. 10.1038/nclimate1191

[B4] Bässler C Müller J Dziock F (2010) Detection of climate-sensitive zones and identification of climate change indicators: A case study from the Bavarian Forest National Park. Folia Geobotanica 45(2): 163–182. 10.1007/s12224-010-9059-4

[B5] Benjamini Y Hochberg Y (1995) Controlling the false discovery rate: A practical and powerful approach to multiple testing. Journal of the Royal Statistical Society. Series B, Statistical Methodology 57(1): 289–300. 10.1111/j.2517-6161.1995.tb02031.x

[B6] Bibelriether H (2004) Nationalpark Bayerischer Wald. Handbuch Naturschutz Und Landschaftspflege 1–7. 10.1002/9783527678471.hbnl1999018

[B7] Bierschenk AM Mueller M Pander J Geist J (2018) Impact of catchment land use on fish community composition in the headwater areas of Elbe, Danube and Main. The Science of the Total Environment 652: 66–74. 10.1016/j.scitotenv.2018.10.218 30359803

[B8] Blacket MJ Robin C Good RT Lee SF Miller AD (2012) Universal primers for fluorescent labelling of PCR fragments—An efficient and cost-effective approach to genotyping by fluorescence. Molecular Ecology Resources 12(3): 456–463. 10.1111/j.1755-0998.2011.03104.x 22268566

[B9] Bojková J Schenková J Horsák M Hájek M (2011) Species richness and composition patterns of clitellate (Annelida) assemblages in the treeless spring fens: The effect of water chemistry and substrate. Hydrobiologia 667(1): 159–171. 10.1007/s10750-011-0634-3

[B10] Breitenmoser-Würsten C Sartori M (1995) Distribution, diversity, life cycle and growth of a mayfly community in a prealpine stream system (Insecta, Ephemeroptera). Hydrobiologia 308(2): 85–101. 10.1007/BF00007393

[B11] Brittain JE (1982) Biology of Mayflies. Annual Review of Entomology 27(1): 119–147. 10.1146/annurev.en.27.010182.001003

[B12] Bunn SE Hughes JM (1997) Dispersal and recruitment in streams: Evidence from genetic studies. Journal of the North American Benthological Society 16(2): 338–346. 10.2307/1468022

[B13] Chapuis M-P Estoup A (2007) Microsatellite null alleles and estimation of population differentiation. Molecular Biology and Evolution 24(3): 621–631. 10.1093/molbev/msl191 17150975

[B14] Chen IC Hill JK Ohlemüller R Roy DB Thomas CD (2011) Rapid range shifts of species associated with high levels of climate warming. Science 333(6045): 1024–1026. 10.1126/science.1206432 21852500

[B15] Collier KJ Smith BJ (1998) Distribution of adult caddisflies (Trichoptera) into forests alongside three New Zealand streams. Hydrobiologia 361(1–3): 53–65. 10.1023/A:1003133208818

[B16] Dangles O (2002) Functional plasticity of benthic macroinvertebrates: Implications for trophic dynamics in acid streams. Canadian Journal of Fisheries and Aquatic Sciences 59(9): 1563–1573. 10.1139/f02-122

[B17] Demandt MH (2010) Temporal changes in genetic diversity of isolated populations of perch and roach. Conservation Genetics 11(1): 249–255. 10.1007/s10592-009-0027-6

[B18] Des Roches S Post DM Turley NE Bailey JK Hendry AP Kinnison MT Schweitzer JA Palkovacs EP (2018) The ecological importance of intraspecific variation. Nature Ecology & Evolution 2(1): 57–64. 10.1038/s41559-017-0402-5 29203921

[B19] Des Roches S Pendleton LH Shapiro B Palkovacs EP (2021) Conserving intraspecific variation for nature’s contributions to people. Nature Ecology & Evolution 5(5): 574–582. 10.1038/s41559-021-01403-5 33649544

[B20] Domisch S Jähnig SC Haase P (2011) Climate-change winners and losers: Stream macroinvertebrates of a submontane region in Central Europe. Freshwater Biology 56(10): 2009–2020. 10.1111/j.1365-2427.2011.02631.x

[B21] Earl DA vonHoldt BM (2012) Structure Harvester: A website and program for visualizing STRUCTURE output and implementing the Evanno method. Conservation Genetics Resources 4(2): 359–361. 10.1007/s12686-011-9548-7

[B22] Eiseler B (2005) Bildbestimmungsschlüssel für die Eintagsfliegenlarven der deutschen Mittelgebirge und des Tieflandes. Lauterbornia 53: 1–112.

[B23] Elbrecht V Feld CK Gies M Hering D Sondermann M Tollrian R Leese F (2014) Genetic diversity and dispersal potential of the stonefly *Dinocras cephalotes* in a central European low mountain range. Freshwater Science 33(1): 181–192. 10.1086/674536

[B24] Evanno G Regnaut S Goudet J (2005) Detecting the number of clusters of individuals using the software structure: A simulation study. Molecular Ecology 14(8): 2611–2620. 10.1111/j.1365-294X.2005.02553.x 15969739

[B25] Excoffier L Lischer HEL (2010) Arlequin suite ver 3.5: A new series of programs to perform population genetics analyses under Linux and Windows. Molecular Ecology Resources 10(3): 564–567. 10.1111/j.1755-0998.2010.02847.x 21565059

[B26] Finn DS Theobald DM Black WC IV Poff NL (2006) Spatial population genetic structure and limited dispersal in a Rocky Mountain alpine stream insect. Molecular Ecology 15(12): 3553–3566. 10.1111/j.1365-294X.2006.03034.x 17032257

[B27] Finn DS Blouin MS Lytle DA (2007) Population genetic structure reveals terrestrial affinities for a headwater stream insect. Freshwater Biology 52(10): 1881–1897. 10.1111/j.1365-2427.2007.01813.x

[B28] Geismar J Sauer J Haase P Nowak C (2011) New microsatellite markers for the assessment of fine-scale dispersal patterns in the endangered montane caddisfly *Drusus discolor*. Conservation Genetics Resources 3(4): 605–607. 10.1007/s12686-011-9414-7

[B29] Geismar J Haase P Nowak C Sauer J Pauls SU (2015) Local population genetic structure of the montane caddisfly *Drusus discolor* is driven by overland dispersal and spatial scaling. Freshwater Biology 60(1): 209–221. 10.1111/fwb.12489

[B30] Gienapp P Teplitsky C Alho JS Mills JA Merilä J (2008) Climate change and evolution: Disentangling environmental and genetic responses. Molecular Ecology 17(1): 167–178. 10.1111/j.1365-294X.2007.03413.x 18173499

[B31] Goudet J (2003) FSTAT Version 2.9.4: A program to estimate and test population genetics parameters. https://www2.unil.ch/popgen/softwares/fstat.htm

[B32] Haidekker A Hering D (2009) Relationship between benthic insects (Ephemeroptera, Plecoptera, Coleoptera, Trichoptera) and temperature in small and medium-sized streams in Germany: A multivariate study. Aquatic Ecology 42(3): 463–481. 10.1007/s10452-007-9097-z

[B33] Hering D Schmidt-Kloiber A Murphy J Lücke S Zamora-Muñoz C López-Rodríguez MJ Huber T Graf W (2009) Potential impact of climate change on aquatic insects: A sensitivity analysis for European caddisflies (Trichoptera) based on distribution patterns and ecological preferences. Aquatic Sciences 71(1): 3–14. 10.1007/s00027-009-9159-5

[B34] Hughes JM Hillyer M Bunn SE (2003) Small-scale patterns of genetic variation in the mayfly *Bungona narilla* (Ephemeroptera: Baetidae) in rainforest streams, south-east Queensland. Freshwater Biology 48(4): 709–717. 10.1046/j.1365-2427.2003.01044.x

[B35] Humpesch UH (1979) Life cycles and growth rates of *Baetis* spp. (Ephemeroptera: Baetidae) in the laboratory and in two stony streams in Austria. Freshwater Biology 9(5): 467–479. 10.1111/j.1365-2427.1979.tb01531.x

[B36] Jensen EL Leigh DM (2022) Using temporal genomics to understand contemporary climate change responses in wildlife. Ecology and Evolution 12(9): e9340. 10.1002/ece3.9340 PMC9481866 36177124

[B37] Kelly LC Bilton DT Rundle SD (2001) Population structure and dispersal in the Canary Island caddisfly *Mesophylax aspersus* (Trichoptera, Limnephilidae). Heredity 86(3): 370–377. 10.1046/j.1365-2540.2001.00839.x 11488974

[B38] Kermek D Pischiutta N Hlebec D Sivec I Kučinić M (2024) Utilising public sequence databases to investigate genetic diversity of stoneflies in Medvednica Nature Park. Biodiversity Data Journal 12: e121398. 10.3897/BDJ.12.e121398 PMC11046089 38680524

[B39] Knispel S Sartori M Brittain JE (2006) Egg development in the mayflies of a Swiss glacial floodplain. Journal of the North American Benthological Society 25(2): 430–443. 10.1899/0887-3593(2006)25[430:EDITMO]2.0.CO;2

[B40] Kovats Z Ciborowski J Corkum L (1996) Inland dispersal of adult aquatic insects. Freshwater Biology 36(2): 265–276. 10.1046/j.1365-2427.1996.00087.x

[B41] Krno I Šporka F Štefková E (2013) The influence of environmental variables on larval growth of stoneflies (Plecoptera) in natural and deforested streams. Biologia 68(5): 497–509. 10.2478/s11756-013-0236-9

[B42] Krno I Žiak M Lánczos T Beracko P Šporka F Thomková K (2021) Stoneflies (Plecoptera) of the Western Carpathians: Does the geological bedrock influence their biodiversity? Biologia 76(12): 3659–3669. 10.1007/s11756-021-00843-5

[B43] Kukula K (1997) The life cycles of three species of Ephemeroptera in two streams in Poland. Hydrobiologia 353(1-3): 193–198. 10.1023/A:1003051104401

[B44] Küttner R Hohmann M Plesky B Voigt H (2008) Zur Verbreitung und Ökologie von *Brachyptera braueri* (Klapálek, 1900) in Mitteldeutschland unter Berücksichtigung weiterer Plecoptera-Arten des zeitigen Frühjahres. Lauterbornia 63: 31–50.

[B45] La Sorte FA Jetz W (2010) Projected range contractions of montane biodiversity under global warming. Proceedings. Biological Sciences 277(1699): 3401–3410. 10.1098/rspb.2010.0612 20534610 PMC2982223

[B46] Laikre L Hoban S Bruford MW Segelbacher G Allendorf FW Gajardo G González Rodríguez A Hedrick PW Heuertz M Hohenlohe PA Jaffé R Johannesson K Liggins L MacDonald AJ Orozco-ter-Wengel P Reusch TBH Rodríguez-Correa H Russo IRM Ryman N Vernesi C (2020) Post-2020 goals overlook genetic diversity. Science 367(6482): 1083–1085. 10.1126/science.abb2748 32139534

[B47] Lavandier P (1982) Evidence of upstream migration by female adults of *Baetis alpinus* Pict. (Ephemeroptera) at high altitude in the Pyrenees. Annales de Limnologie 18(1): 55–59. 10.1051/limn/1982015

[B48] Lavandier P (1988) Semivoltinisme dans des populations de haute montagne de *Baetis alpinus* Pictet (Ephemeroptera). Bulletin de la Société d’Histoire Naturelle de Toulouse 124: 61–64.

[B49] Lavandier P (1992) Larval production and drift of *Drusus discolor* (Trichoptera, Limnephilidae) in a high mountain stream in the Pyrénées (France). Archiv für Hydrobiologie 83(1): 83–96. 10.1127/archiv-hydrobiol/125/1992/83

[B50] Lehrian S Pauls SU Haase P (2009) Contrasting patterns of population structure in the montane caddisflies *Hydropsyche tenuis* and *Drusus discolor* in the Central European highlands. Freshwater Biology 54(2): 283–295. 10.1111/j.1365-2427.2008.02107.x

[B51] Leigh DM Hendry AP Vázquez-Domínguez E Friesen VL (2019) Estimated six per cent loss of genetic variation in wild populations since the industrial revolution. Evolutionary Applications 12(8): 1505–1512. 10.1111/eva.12810 31462910 PMC6708419

[B52] Leys M Keller I Räsänen K Gattolliat JL Robinson CT (2016) Distribution and population genetic variation of cryptic species of the Alpine mayfly *Baetis alpinus* (Ephemeroptera: Baetidae) in the Central Alps. BMC Evolutionary Biology 16(1): 77. 10.1186/s12862-016-0643-y 27068234 PMC4828801

[B53] Leys M Keller I Robinson CT Räsänen K (2017) Cryptic lineages of a common alpine mayfly show strong life-history divergence. Molecular Ecology 26(6): 1670–1686. 10.1111/mec.14026 28099770

[B54] Lods-Crozet B Castella E Cambin D Ilg C Knispel S Mayor-Simeant H (2001) Macroinvertebrate community structure in relation to environmental variables in a Swiss glacial stream. Freshwater Biology 46(12): 1641–1661. 10.1046/j.1365-2427.2001.00850.x

[B55] López-Rodríguez MJ De Figueroa JT Fenoglio S Bo T Alba-Tercedor J (2009) Life strategies of 3 Perlodidae species (Plecoptera) in a Mediterranean seasonal stream in southern Europe. Journal of the North American Benthological Society 28(3): 611–625. 10.1899/08-105.1

[B56] Lyam PT Duque-Lazo J Hauenschild F Schnitzler J Muellner-Riehl AN Greve M Ndangalasi H Myburgh A Durka W (2022) Climate change will disproportionately affect the most genetically diverse lineages of a widespread African tree species. Scientific Reports 12(1): 7035. 10.1038/s41598-022-11182-z 35488120 PMC9054768

[B57] Madsen BL Bengtson J Butz I (1973) Observations on upstream migration by imagines of some Plecoptera and Ephemeroptera. Limnology and Oceanography 18(4): 678–681. 10.4319/lo.1973.18.4.0678

[B58] Malison RL Hand BK Winter E Giersch JJ Amish SJ Whited D Stanford JA Luikart G (2022) Landscape connectivity and genetic structure in a mainstem and a tributary stonefly (Plecoptera) species using a novel reference genome. The Journal of Heredity 113(4): 453–471. 10.1093/jhered/esac025 35569065

[B59] McCain CM Grytnes JA (2010) Elevational gradients in species richness. Encyclopedia of Life Sciences. 10.1002/9780470015902.a0022548

[B60] Monaghan MT Spaak P Robinson CT Ward JV (2001) Genetic differentiation of *Baetis alpinus* Pictet (Ephemeroptera: Baetidae) in fragmented alpine streams. Heredity 86(4): 395–403. 10.1046/j.1365-2540.2001.00843.x 11520339

[B61] Monaghan MT Spaak P Robinson CT Ward JV (2002) Population genetic structure of 3 alpine stream insects: Influences of gene flow, demographics, and habitat fragmentation. Journal of the North American Benthological Society 21(1): 114–131. 10.2307/1468304

[B62] Moussalli A Moritz C Williams SE Carnaval AC (2009) Variable responses of skinks to a common history of rainforest fluctuation: Concordance between phylogeography and palaeo‐distribution models. Molecular Ecology 18(3): 483–499. 10.1111/j.1365-294X.2008.04035.x 19161469

[B63] NPBW (2023) Der Nationalpark Bayerischer Wald im Porträt. https://www.nationalpark-bayerischer-wald.bayern.de/ueber_uns/steckbrief/index.htm

[B64] Pauls SU Lumbsch HT Haase P (2006) Phylogeography of the montane caddisfly *Drusus discolor*: Evidence for multiple refugia and periglacial survival. Molecular Ecology 15(8): 2153–2169. 10.1111/j.1365-294X.2006.02916.x 16780432

[B65] Pauls SU Feldheim KA Haase P (2007) Isolation and characterization of microsatellite markers in the caddisfly *Drusus discolor* (Trichoptera: Limnephilidae). Molecular Ecology Notes 7(1): 150–152. 10.1111/j.1471-8286.2006.01562.x

[B66] Pauls SU Theissinger K Ujvarosi L Balint M Haase P (2009) Patterns of population structure in two closely related, partially sympatric caddisflies in Eastern Europe: historic introgression, limited dispersal, and cryptic diversity. Journal of the North American Benthological Society 28(3): 517–536. 10.1899/08-100.1

[B67] Pauls SU Nowak C Bálint M Pfenninger M (2013) The impact of global climate change on genetic diversity within populations and species. Molecular Ecology 22(4): 925–946. 10.1111/mec.12152 23279006

[B68] Pritchard JK Stephens M Donnelly P (2000) Inference of population structure using multilocus genotype data. Genetics 155(2): 945–959. 10.1093/genetics/155.2.945 10835412 PMC1461096

[B69] Puechmaille SJ (2016) The program structure does not reliably recover the correct population structure when sampling is uneven: Subsampling and new estimators alleviate the problem. Molecular Ecology Resources 16(3): 608–627. 10.1111/1755-0998.12512 26856252

[B70] Reed DH Frankham R (2003) Correlation between fitness and genetic diversity. Conservation Biology 17(1): 230–237. 10.1046/j.1523-1739.2003.01236.x

[B71] Rosenberg NA (2004) DISTRUCT: A program for the graphical display of population structure. Molecular Ecology Notes 4(1): 137–138. 10.1046/j.1471-8286.2003.00566.x

[B72] Rutschmann S Detering H Simon S Funk DH Gattolliat JL Hughes SJ Raposeiro PM DeSalle R Sartori M Monaghan MT (2017) Colonization and diversification of aquatic insects on three Macaronesian archipelagos using 59 nuclear loci derived from a draft genome. Molecular Phylogenetics and Evolution 107: 27–38. 10.1016/j.ympev.2016.10.007 27742475

[B73] Sauer J Domisch S Nowak C Haase P (2011) Low mountain ranges: Summit traps for montane freshwater species under climate change. Biodiversity and Conservation 20(13): 3133–3146. 10.1007/s10531-011-0140-y

[B74] Schmidt SK Hughes JM Bunn SE (1995) Gene flow among conspecific populations of *Baetis* sp. (Ephemeroptera): Adult flight and larval drift. Journal of the North American Benthological Society 14(1): 147–157. 10.2307/1467730

[B75] Schröder O Schneider JV Schell T Seifert L Pauls SU (2022) Population genetic structure and connectivity in three montane freshwater invertebrate species (Ephemeroptera, Plecoptera, Amphipoda) with differing life cycles and dispersal capabilities. Freshwater Biology 67(3): 461–472. 10.1111/fwb.13854

[B76] Sode A Wiberg-Larsen P (1993) Dispersal of adult Trichoptera at a Danish forest brook. Freshwater Biology 30(3): 439–446. 10.1111/j.1365-2427.1993.tb00827.x

[B77] Taubmann J Theissinger K Feldheim KA Laube I Graf W Haase P Johannsen J Pauls SU (2011) Modelling range shifts and assessing genetic diversity distribution of the montane aquatic mayfly *Ameletus inopinatus* in Europe under climate change scenarios. Conservation Genetics 12(2): 503–515. 10.1007/s10592-010-0157-x

[B78] Thomas AGB (1975) Éphéméroptères du Sud-Ouest de la France. I: Migration d’imagos à haute altitude. Annales de Limnologie 11(1): 47–66. 10.1051/limn/1975016

[B79] Truett GE Heeger P Mynatt RL Truett AA Walker JA Warman ML (2000) Preparation of PCR-quality mouse genomic DNA with hot sodium hydroxide and tris (HotSHOT). BioTechniques 29(1): 52–54. 10.2144/00291bm09 10907076

[B80] Tyufekchieva V Kalcheva H Vidinova Y Yaneva I Stoyanova T Ljubomirov T (2012) Distribution and ecology of Taeniopterygidae (Insecta: Plecoptera) in Bulgaria. Acta Zoologica Bulgarica 65: 89–100. 10.2478/v10210-011-0028-1

[B81] Ward JV Stanford JA (1982) Thermal responses in the evolutionary ecology of aquatic insects. Annual Review of Entomology 27(1): 97–117. 10.1146/annurev.en.27.010182.000525

[B82] Waringer J Graf W (2011) Atlas der mitteleuropäischen Köcherfliegenlarven/Atlas of Central European Trichoptera Larvae. Erik Mauch Verlag, Dinkelscherben, 1–468.

[B83] Zwick P (2004) Key to the west Palaearctic genera of stoneflies (Plecoptera) in the larval stage. Limnologica 34(4): 315–348. 10.1016/S0075-9511(04)80004-5

