# Supplementary material for: ﻿Temporal monitoring of genetic diversity in aquatic insects: a pilot study in the Bavarian Forest National Park
Source: Zookeys. 2025 Dec 10;1263:499–518. doi: 10.3897/zookeys.1263.147797 (PMC12712642; doi:10.3897/zookeys.1263.147797)
Supplement: Supplementary material 1 — Supplementary information [file zookeys-1263-499_article-147797__-s001.docx]

**Supplementary Materials**

| **Temporal monitoring of genetic diversity in aquatic insects: a pilot study in the Bavarian Forest National Park**  Jan Simon Stark, Oskar Schröder, Jörg Müller, Linda Seifert, Steffen U Pauls  Table 1. Geographical coordinates and elevation of sampling sites at the Kolbersbach (KB), Kleine Ohe (KO), and Grosse Deffernik (GD) in the Bavarian Forest National Park. | | | |
| --- | --- | --- | --- |
| Sampling site | Decimal Degrees (DD) | Exact elevation (m a.s.l.) | Elevation gradient category (m a.s.l.) |
| KB1 | 49.0917° N, 13.3131° E | 1098 | 1100 |
| KB3 | 49.0765° N, 13.3146° E | 845 | 900 |
| KB4 | 49.0711° N, 13.3110° E | 786 | 800 |
| KB5 | 49.0614° N, 13.2986° E | 700 | 700 |
| KO1 | 48.9448° N, 13.4715° E | 1056 | 1100 |
| KO2 | 48.9410° N, 13.4698° E | 992 | 1000 |
| KO3 | 48.9380° N, 13.4671° E | 911 | 900 |
| KO4 | 48.9326° N, 13.4524° E | 801 | 800 |
| KO5 | 48.9094° N, 13.4456° E | 706 | 700 |
| GD1 | 49.1082° N, 13.2983° E | 1080 | 1100 |
| GD2 | 49.1105° N, 13.2916° E | 991 | 1000 |
| GD3 | 49.1138° N, 13.2785° E | 892 | 900 |
| GD4 | 49.1179° N, 13.2570° E | 787 | 800 |
| GD5 | 49.1096° N, 13.2360° E | 698 | 700 |

| Table 2. Microsatellite loci and primers for *Baetis alpinus* used in this study. | | | | | |
| --- | --- | --- | --- | --- | --- |
| Locus ID | Forward and reverse primer sequences (5’ - 3’) | | Repeat motif | Range (bp) | Dye |
| Ba_c1 | TGCATCGAGGGACACAATCG; TAAAGAGAAAAACTGGTCCTCGC | CA | | 109-228 | Atto 550 |
| Ba_c2 | TCAGGAAACCACTTCCCGAG; GCATGTCCACGCCATGTATC | GCG | | 195-217 | HEX |
| Ba_c3 | TATCCAATCCCACGCACTCC; GAAGCGGACGTGGTTATGTG | AC | | 206-253 | Atto 550 |
| Ba_c4 | TGGCTCCTCCAAGTACAACG; CGTACGCACACACTGACATC | AC | | 224-267 | Atto 565 |
| Ba_g1 | TGCATCGAGGGACACAATCG; TAAAGAGAAAAACTGGTCCTCGC | AC | | 84-159 | FAM |
| Ba_g2 | TGAGCAGATTACCACCACCG; GCGATGGGTTCGGAAATGAG | GT | | 158-241 | FAM |
| Ba_g4 | TTGAGCATCGCCGAATCAAC; TTCGCTCTGGTGTATGGGTG | GA | | 115-246 | HEX |
| Ba_g5 | TGTTCGTCTCTATCCCGCAG; TGTAACTCTCTCGGTGATGTC | AC | | 187-241 | FAM |
| Ba_g6 | GCGCGTTTTTAACTCAAATGAAAG; TAGGCAGGCAAGGCGGAC | TG | | 109-155 | HEX |

| Table 3. Microsatellite loci and primers for *Brachyptera seticornis* used in this study. | | | | | |
| --- | --- | --- | --- | --- | --- |
| Locus ID | Forward and reverse primer sequences (5’ - 3’) | | Repeat motif | Range (bp) | Dye |
| BS_11 | GGACAAAGATCCATGAGAGTA; GGACACAAGCCTTAGATGTAT | ATA | | 183-229 | Atto 550 |
| BS_16 | GTTTTGGATAGTGGAGATGGA; AGACCAAAGTTTGATTTCTGC | TAT | | 206-227 | Atto 565 |
| BS_18 | ACAACGATGTCATTTCATCAC; CAAAACTTCGTCAATCCAGTT | TGT | | 198-258 | HEX |
| BS_34 | CGAAGACAAAGAAGAAGATGG; TTCATCTTCTTCCAACTCCTC | AAG | | 274-312 | HEX |
| BS_35 | CCATCAATTTCAATAAATGCGT; TTTATCACCATCCTCATCACC | TGA | | 268-308 | Atto 550 |
| BS_36 | ACATTCTAAGAAGAGATGCGA; CCAATAATTGAGTGGCAAGAG | ATT | | 303-324 | Atto 565 |
| BS_43 | TGAGGTCATTTGTTGGTAAGT; CAAAATATGGCCACAACTCAA | TAA | | 295-318 | Atto 550 |
| BS_52 | GAAAGTCTGTTGCATTTGACT; TGTGTAACATGTCTGAAGTTAG | ATA | | 309-338 | Atto 565 |
| BS_53 | CCTAGGCCTATAGTTTCTTGG; TATACACAAGTCTGATGCAGG | ATA | | 303-324 | FAM |

| Table 4. Microsatellite loci and primers for *Drusus discolor* used in this study. | | | | | |
| --- | --- | --- | --- | --- | --- |
| Locus ID | Forward and reverse primer sequences (5’ - 3’) | | Repeat motif | Range (bp) | Dye |
| DD_03 | TGGAACAATCGAAGTTATTTAGGA; GCTGCTCAAGATGAACACCA | GTTA | | 176-237 | HEX |
| DD_05 | ATCTCCCAAATGGTTTCGTG; TTTGCGGGGGTGTTAAATAG | ATG | | 145-158 | Atto 565 |
| DD_12 | GCGCCAAATGTCAGATGTAAGA; CGCTGTCTGGATCAGACGTA | CATA | | 214-239 | Atto 550 |
| DD_17 | ATCGGAAAAATGGTCAAGCA; GTGTCTTCGATCGGACTAGGAGGAAAC | GTAT | | 225-274 | Atto 565 |
| DD_31 | TTCCGGAACGAATAATGCTC; GAATGGAGACCACTCGGATG | GATT | | 186-219 | HEX |
| DD_52 | AATGGATTCTTTGCATTTGG; CGGCAACATACCACATAACA | GTTT | | 212-272 | Atto 550 |
| DD_59 | TCTCGCATTTCGTTGTTATCTG; ACACATTGTGCCATCGGTAA | AT | | 144-148 | Atto 550 |
| DD_61 | TCCAAAAATTACGACGTTTGC; TCCAAAAATTACGACGTTTGC | AT | | 203-237 | Atto 565 |
| DD_66 | AGTCGACACCCCATCGTAAA; CTGCCATATCCACGAGCTACT | TTTTA | | 219-238 | Atto 565 |

| Table 5. Genetic diversity analysis of *B. alpinus* (*n* = 735) for nine microsatellite loci among all sampling sites in the Bavarian Forest National Park. Metrics include (H_o_) for observed heterozygosity, (H_e_) for expected heterozygosity, (F_ST_) for heterozygosity deficiency among the loci, and (F_IS_) for deficiency within each microsatellite locus. The table also provides (A_R_) for allelic richness and (A_T_) for the total number of alleles. All calculations were based on Nei’s estimators of gene diversities and differentiation (Nei 1987). | | | | | | |
| --- | --- | --- | --- | --- | --- | --- |
| Locus name | H_o_ | H_e_ | F_ST_ | F_IS_ | A_R_ | A_T_ |
| Ba_c1 | 0.77 | 0.78 | 0.05 | 0.02 | 7.73 | 15 |
| Ba_c2 | 0.68 | 0.75 | 0.00 | 0.09 | 5.35 | 7 |
| Ba_g5 | 0.30 | 0.50 | 0.03 | 0.40 | 4.30 | 7 |
| Ba_c4 | 0.75 | 0.82 | 0.02 | 0.08 | 7.92 | 17 |
| Ba_g1 | 0.68 | 0.74 | 0.02 | 0.09 | 7.15 | 13 |
| Ba_g6 | 0.59 | 0.62 | 0.03 | 0.05 | 5.11 | 11 |
| Ba_c3 | 0.41 | 0.81 | 0.02 | 0.49 | 8.17 | 17 |
| Ba_g2 | 0.54 | 0.79 | 0.02 | 0.32 | 7.41 | 14 |
| Ba_g4 | 0.75 | 0.85 | 0.01 | 0.12 | 9.11 | 19 |
| Mean | 0.61 ± 0.15 | 0.74 ± 0.10 | 0.02 ± 0.01 | 0.18 ± 0.16 | 6.92 ± 1.52 | 13.33 ± 4.06 |

| Table 6. AMOVA of *B. alpinus* among streams, among sites within streams, and within sites across years in the Bavarian Forest National Park. Asterisks (*) indicate significance (p < 0.05). | | | |
| --- | --- | --- | --- |
| Source of variation | Sum of squares | Variance components | Percentage variation |
| Among streams | 33.720 | 0.00946 | 0.27916 |
| Among sites within streams | 87.955 | 0.06679 | 1.97099* |
| Within sites | 4514.285 | 3.31264 | 97.74985 |
| Total | 4635.959 | 3.40957 | 100 |

| Table 7. Pairwise F_ST_ estimates for *B. alpinus* among each sampling site. Asterisks (*) indicate significance after FDR (Benjamini and Hochberg 1995) correction of p-values (p < 0.05). | | | | | | | | | | | |
| --- | --- | --- | --- | --- | --- | --- | --- | --- | --- | --- | --- |
|  | KB1 | KB3 | KB4 | KB5 | KO3 | KO5 | GD1 | GD2 | GD3 | GD4 | GD5 |
| KB1 |  |  |  |  |  |  |  |  |  |  |  |
| KB3 | 0.01619* |  |  |  |  |  |  |  |  |  |  |
| KB4 | 0.05725* | 0.02031* |  |  |  |  |  |  |  |  |  |
| KB5 | 0.04182* | 0.05032* | 0.08752* |  |  |  |  |  |  |  |  |
| KO3 | 0.02020* | 0.02059* | 0.04806* | 0.00785 |  |  |  |  |  |  |  |
| KO5 | 0.02902* | 0.03500* | 0.07759* | 0.00460 | 0.01504 |  |  |  |  |  |  |
| GD1 | 0.01966* | 0.02194* | 0.05070* | 0.01456* | -0.00162 | 0.00697* |  |  |  |  |  |
| GD2 | 0.02702* | 0.03267* | 0.07232* | 0.00488 | 0.00438 | -0.00364 | 0.00280 |  |  |  |  |
| GD3 | 0.02127* | 0.03148* | 0.06817* | 0.00348 | 0.00201 | -0.00033 | 0.00635* | 0.00187 |  |  |  |
| GD4 | 0.01491* | 0.02520* | 0.04630* | 0.01504* | 0.00695 | 0.00960* | 0.01276* | 0.00971* | 0.00650* |  |  |
| GD5 | 0.01906* | 0.01062* | 0.02384* | 0.03208* | 0.00348 | 0.01854* | 0.00583* | 0.02016* | 0.01511* | 0.01222* |  |

| Table 8. AMOVA of *B. alpinus* between years across all sampling sites in the Bavarian Forest National Park from 2016-2023. Asterisks (*) indicate significance (p < 0.05). | | | |
| --- | --- | --- | --- |
| Source of variation | Sum of squares | Variance components | Percentage variation |
| Among years | 43.613 | 0.02000 | 0.59163* |
| Within years | 4598.140 | 3.36100 | 99.40837 |
| Total | 4641.753 | 3.38100 | 100 |

| Table 9. Pairwise F_ST_ estimates for *Baetis alpinus* among all sampling sites from 2016-2023. Asterisks (*) indicate significance after FDR (Benjamini and Hochberg 1995) corrections of p-values (p < 0.05). | | | | | | | |
| --- | --- | --- | --- | --- | --- | --- | --- |
|  | 2016 | 2018 | 2019 | 2020 | 2021 | 2022 | 2023 |
| 2016 |  |  |  |  |  |  |  |
| 2018 | 0.00700* |  |  |  |  |  |  |
| 2019 | 0.01287* | -0.00068 |  |  |  |  |  |
| 2020 | 0.01191* | 0.00717* | 0.00200 |  |  |  |  |
| 2021 | 0.00533 | 0.00211 | -0.00074 | -0.00764 |  |  |  |
| 2022 | 0.00954* | 0.00460* | -0.00044 | 0.00116 | -0.00371 |  |  |
| 2023 | 0.01591* | 0.00615* | 0.00717* | 0.00207 | 0.00057 | 0.00226 |  |

| Table 10. Genetic diversity analysis of ***Brachyptera*** *seticornis* (*n* = 295) for nine microsatellite loci among all sampling sites in the Bavarian Forest National Park. Metrics include (H_o_) for observed heterozygosity, (H_e_) for expected heterozygosity, (F_ST_) for heterozygosity deficiency among the loci, and (F_IS_) for deficiency within each microsatellite locus. The table also provides (A_R_) for allelic richness and (A_T_) for the total number of alleles. All calculations were based on Nei’s estimators of gene diversities and differentiation (Nei 1987). | | | | | | |
| --- | --- | --- | --- | --- | --- | --- |
| Locus name | H_o_ | H_s_ | F_ST_ | F_IS_ | A_R_ | A_T_ |
| BS_18 | 0.75 | 0.75 | 0.00 | 0.00 | 5.09 | 22 |
| BS_43 | 0.46 | 0.51 | 0.03 | 0.09 | 3.24 | 7 |
| BS_52 | 0.60 | 0.61 | 0.01 | 0.02 | 3.72 | 8 |
| BS_11 | 0.67 | 0.71 | 0.00 | 0.06 | 4.45 | 17 |
| BS_16 | 0.32 | 0.66 | -0.01 | 0.51 | 3.14 | 5 |
| BS_53 | 0.32 | 0.32 | 0.02 | 0.00 | 2.39 | 8 |
| BS_34 | 0.25 | 0.24 | 0.05 | -0.04 | 2.01 | 5 |
| BS_35 | 0.11 | 0.18 | -0.01 | 0.36 | 1.81 | 6 |
| BS_36 | 0.42 | 0.50 | -0.03 | 0.16 | 2.06 | 4 |
| Mean | 0.43 ± 0.20 | 0.50 ± 0.20 | 0.01 ± 0.02 | 0.13 ± 0.18 | 3.10 ± 1.09 | 9.11 ± 5.82 |

| Table 11. Pairwise F_ST_ estimates for ***Brachyptera*** *seticornis* among all sampling sites from 2018-2023. Asterisks (*) indicate significance after FDR (Benjamini and Hochberg 1995) correction of p-values (p < 0.05). | | | | | | |
| --- | --- | --- | --- | --- | --- | --- |
|  | 2018 | 2019 | 2020 | 2021 | 2022 | 2023 |
| 2018 |  |  |  |  |  |  |
| 2019 | -0.00394 |  |  |  |  |  |
| 2020 | 0.01741* | 0.02510 |  |  |  |  |
| 2021 | -0.00902 | 0.00052 | 0.02059 |  |  |  |
| 2022 | 0.00436 | 0.00233 | 0.01245 | -0.01523 |  |  |
| 2023 | -0.00304 | 0.00135 | 0.01993* | 0.01172 | 0.00728 |  |

| Table 12. Pairwise F_ST_ estimates for  ***Brachyptera*** *seticornis* among each sampling site. Asterisks (*) indicate significance after FDR (Benjamini and Hochberg 1995) correction of p-values (p < 0.05). No values were significant. | | | | | | | | | | | |
| --- | --- | --- | --- | --- | --- | --- | --- | --- | --- | --- | --- |
|  | KB1 | KB3 | KB5 | KO1 | KO2 | KO3 | KO5 | GD1 | GD2 | GD3 | GD5 |
| KB1 |  |  |  |  |  |  |  |  |  |  |  |
| KB3 | 0.00695 |  |  |  |  |  |  |  |  |  |  |
| KB5 | 0.04447 | 0.00085 |  |  |  |  |  |  |  |  |  |
| KO1 | -0.01146 | 0.00080 | 0.02861 |  |  |  |  |  |  |  |  |
| KO2 | -0.00243 | 0.00729 | 0.02804 | -0.01208 |  |  |  |  |  |  |  |
| KO3 | -0.00190 | -0.00795 | -0.00286 | 0.00403 | 0.00873 |  |  |  |  |  |  |
| KO5 | 0.00730 | 0.00208 | 0.01374 | 0.00713 | 0.02266 | -0.03346 |  |  |  |  |  |
| GD1 | 0.00343 | -0.00224 | 0.03912 | -0.00776 | 0.00945 | -0.01041 | 0.00460 |  |  |  |  |
| GD2 | 0.00505 | 0.00866 | 0.02410 | -0.02074 | 0.00409 | -0.01214 | 0.01251 | 0.00200 |  |  |  |
| GD3 | -0.00060 | 0.00738 | 0.03583 | 0.00010 | 0.00256 | 0.00010 | 0.00952 | 0.00981 | 0.02143 |  |  |
| GD5 | 0.00300 | 0.01168 | 0.05126 | 0.00346 | 0.00200 | 0.00896 | 0.00860 | 0.00707 | 0.02355 | 0.00248 |  |

| Table 13. AMOVA of ***Brachyptera***  *seticornis* for all streams in the Bavarian Forest National Park from 2016-2023. Asterisks (*) indicate significance (p < 0.05). | | | |
| --- | --- | --- | --- |
| Source of variation | Sum of squares | Variance components | Percentage variation |
| Among years | 17.203 | 0.01706 | 0.85414* |
| Within years | 1073.760 | 1.98067 | 99.14586 |
| Total | 1090.963 | 1.99773 | 100 |

| Table 14. AMOVA of ***Brachyptera*** *seticornis* among streams, among sites within streams, and within sites in the Bavarian Forest National Park. Asterisks (*) indicate significance (p < 0.05). | | | |
| --- | --- | --- | --- |
| Source of variation | Sum of squares | Variance components | Percentage variation |
| Among streams | 5.101 | -0.00196 | -0.08747 |
| Among sites within streams | 22.406 | 0.01141 | 0.50913 |
| Within sites | 1175.299 | 2.23205 | 99.57834 |
| Total | 1202.806 | 2.24150 | 100 |

| Table 15. Genetic diversity analysis of *Drusus discolor* (*n* = 193) for nine microsatellite loci among all sampling sites in the Bavarian Forest National Park. Metrics include (H_o_) for observed heterozygosity, (H_e_) for expected heterozygosity, (F_ST_) for heterozygosity deficiency among the loci, and (F_IS_) for deficiency within each microsatellite locus. The table also provides (A_R_) for allelic richness and (A_T_) for the total number of alleles. All calculations were based on Nei’s estimators of gene diversities and differentiation (Nei 1987). | | | | | | |
| --- | --- | --- | --- | --- | --- | --- |
| Locus name | H_o_ | H_s_ | F_ST_ | F_IS_ | A_R_ | A_T_ |
| DD_05 | 0.00 | 0.00 | -0.06 | 0.07 | 1.01 | 2 |
| DD_31 | 0.77 | 0.77 | 0.07 | -0.01 | 3.27 | 14 |
| DD_61 | 0.28 | 0.77 | -0.13 | 0.64 | 2.46 | 6 |
| DD_12 | 0.64 | 0.69 | -0.05 | 0.07 | 2.45 | 10 |
| DD_17 | 0.76 | 0.75 | 0.05 | -0.01 | 2.88 | 17 |
| DD_59 | 0.34 | 0.30 | 0.22 | -0.12 | 1.51 | 9 |
| DD_03 | 0.55 | 0.61 | -0.02 | 0.10 | 2.29 | 11 |
| DD_52 | 0.82 | 0.89 | 0.01 | 0.09 | 3.26 | 32 |
| DD_66 | 0.33 | 0.52 | -0.07 | 0.37 | 1.98 | 5 |
| Mean | 0.50 ± 0.26 | 0.59 ± 0.26 | 0.00 ± 0.10 | 0.13 ± 0.22 | 2.35 ± 0.76 | 11.78 ± 8.86 |

| Table 16. Pairwise F_ST_ estimates for *Drusus discolor* among all sampling sites from 2016-2023. Asterisks (*) indicate significance after FDR (Benjamini and Hochberg 1995) correction of p-values (p < 0.05). | | | | | | | |
| --- | --- | --- | --- | --- | --- | --- | --- |
|  | 2016 | 2018 | 2019 | 2020 | 2021 | 2022 | 2023 |
| 2016 |  |  |  |  |  |  |  |
| 2018 | 0.00035 |  |  |  |  |  |  |
| 2019 | 0.03786* | 0.03646* |  |  |  |  |  |
| 2020 | 0.00529 | 0.00909 | -0.01208 |  |  |  |  |
| 2021 | -0.15823 | -0.14904 | -0.15698 | -0.10113 |  |  |  |
| 2022 | 0.03020* | 0.03325* | 0.00470 | -0.01102 | -0.16783 |  |  |
| 2023 | 0.01749 | 0.02203* | 0.00893 | -0.00713 | -0.10767 | -0.00287 |  |

| Table 17. Pairwise F_ST_ estimates for *Drusus discolor* among each sampling site. Asterisks (*) indicate significance after FDR (Benjamini and Hochberg 1995) correction of p-values (p < 0.05). | | | | | | |
| --- | --- | --- | --- | --- | --- | --- |
|  | KB1 | KB3 | KO1 | KO5 | GD1 | GD3 |
| KB1 |  |  |  |  |  |  |
| KB3 | 0.06126 |  |  |  |  |  |
| KO1 | 0.00186 | 0.06640 |  |  |  |  |
| KO5 | -0.02299 | 0.05747 | -0.00242 |  |  |  |
| GD1 | 0.01955 | 0.07316 | 0.00004 | 0.03413 |  |  |
| GD3 | 0.05242* | 0.13965* | 0.02213 | 0.06145 | 0.02079 |  |

| Table 18. AMOVA of *Drusus discolor* for all streams in the Bavarian Forest National Park from 2016-2023. Asterisks (*) indicate significance (p < 0.05). | | | |
| --- | --- | --- | --- |
| Source of variation | Sum of squares | Variance components | Percentage variation |
| Among years | 16.198 | 0.01804 | 0.99128* |
| Within years | 650.193 | 1.80206 | 99.00872 |
| Total | 666.391 | 1.82010 | 100 |

| Table 19. AMOVA of *Drusus discolor* among streams, among sites within streams, and within sites in the Bavarian Forest National Park. Asterisks (*) indicate significance (p < 0.05). | | | |
| --- | --- | --- | --- |
| Source of variation | Sum of squares | Variance components | Percentage variation |
| Among streams | 8.499 | 0.00553 | 0.21280 |
| Among sites within streams | 9.336 | 0.02125 | 0.81723 |
| Within sites | 902.503 | 2.57404 | 98.96996 |
| Total | 920.338 | 2.60083 | 100 |
